# The Tie2-agonist Vasculotide rescues mice from influenza virus infection

**DOI:** 10.1038/srep11030

**Published:** 2015-06-05

**Authors:** Michael G. Sugiyama, Susan M. Armstrong, Changsen Wang, David Hwang, Howard Leong-Poi, Andrew Advani, Suzanne Advani, Haibo Zhang, Katalin Szaszi, Arata Tabuchi, Wolfgang M. Kuebler, Paul Van Slyke, Dan J. Dumont, Warren L. Lee

**Affiliations:** 1 Keenan Research Centre for Biomedical Science, St. Michael’s Hospital, Toronto, ON; 2 Department of Laboratory Medicine and Pathobiology, University of Toronto; 3 Department of Pathology, Toronto General Hospital, University Health Network, Toronto, ON; 4 Sunnybrook Research Institute, Sunnybrook Health Sciences Centre, Toronto, ON; 5 Interdepartmental Division of Critical Care and Department of Medicine, University of Toronto.

## Abstract

Seasonal influenza virus infections cause hundreds of thousands of deaths annually while viral mutation raises the threat of a novel pandemic strain. Antiviral drugs exhibit limited efficacy unless administered early and may induce viral resistance. Thus, targeting the host response directly has been proposed as a novel therapeutic strategy with the added potential benefit of not eliciting viral resistance. Severe influenza virus infections are complicated by respiratory failure due to the development of lung microvascular leak and acute lung injury. We hypothesized that enhancing lung endothelial barrier integrity could improve the outcome. Here we demonstrate that the Tie2-agonist tetrameric peptide Vasculotide improves survival in murine models of severe influenza, even if administered as late as 72 hours after infection; the benefit was observed using three strains of the virus and two strains of mice. The effect required Tie2, was independent of viral replication and did not impair lung neutrophil recruitment. Administration of the drug decreased lung edema, arterial hypoxemia and lung endothelial apoptosis; importantly, Vasculotide is inexpensive to produce, is chemically stable and is unrelated to any Tie2 ligands. Thus, Vasculotide may represent a novel and practical therapy for severe infections with influenza.

Whether as an agent of pandemics or as a seasonal pathogen, the influenza virus exacts a heavy toll on global public health. Despite vaccination programs and antiviral drugs, seasonal influenza alone causes millions of cases of severe illness and hundreds of thousands of deaths annually^1^,^2^. There are concerns that ongoing mutation will lead to a novel strain of the virus that is both highly transmissible *and* highly virulent, as occurred in the 1918 pandemic leading to the deaths of 50 million people^3^. Existing treatments for the virus are inadequate: antiviral drugs are not completely effective at reducing mortality^4^,^5^ and exhibit declining efficacy unless given at the time of infection, a problematic limitation since the time of infection is usually unknown^6^,^7^. There is also the problem of antiviral resistance; of the two classes of drugs approved for use against influenza, one is largely ineffective due to widespread resistance, while sporadic cases of resistance to the other have been reported^8^,^9^. Thus, there is a need for novel therapies; those that target the host rather than the pathogen may be ideal as they should be less susceptible to viral resistance.

Most deaths from influenza virus infection occur due to pulmonary complications, in particular the development of acute respiratory distress syndrome (ARDS)^10^,^11^, a potentially fatal syndrome of pulmonary edema that occurs due to increased permeability of the lung microvasculature^12^,^13^. Blood vessels in the lung are lined by a continuous layer of endothelium; thus, loss of endothelial barrier integrity is a prerequisite for ARDS.

The fact that mortality persists despite antiviral therapy suggests that elements of the host response may be maladaptive^14^,^15^. Given the prominence of ARDS in severe infections, we hypothesized that enhancement of lung endothelial barrier function might improve survival. We tested the novel Tie2-agonist tetrameric peptide, Vasculotide; this peptide has no sequence homology with any endogenous ligands, is easy to produce and is chemically stable^16^. Here we report that administration of Vasculotide significantly improves survival from influenza virus infection, even when started several days post-infection.

## Results and Discussion

**Figure 1:**
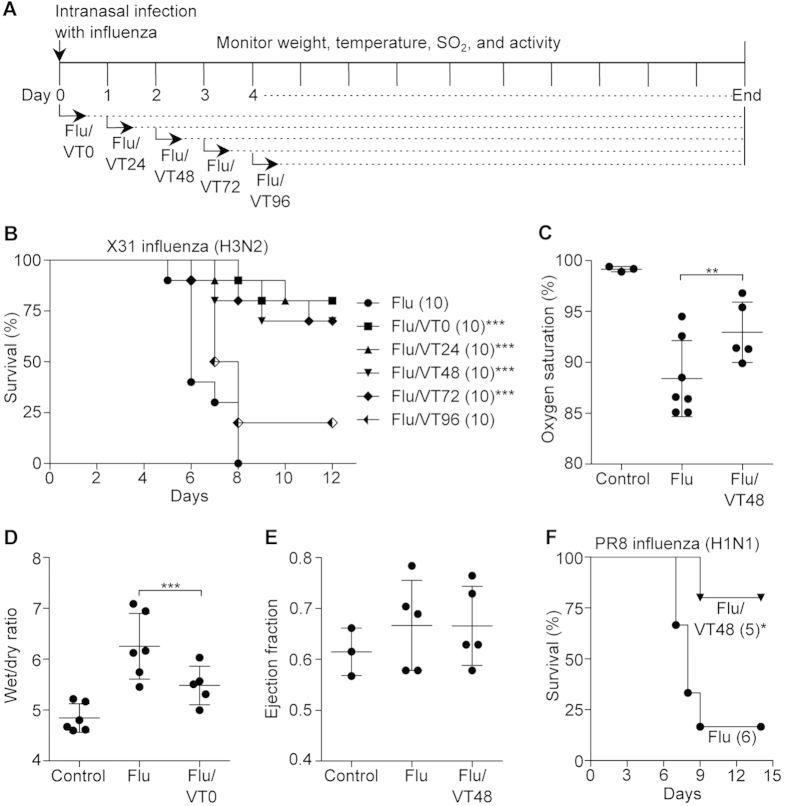
Vasculotide (VT) protects and rescues mice from severe influenza virus infection. **(a)** Basic experimental protocol used to test VT in mice infected with influenza virus. Arrows indicate the time after infection (hours) at which daily administration of VT was begun. C57BL/6J mice were infected with mouse-adapted influenza, strain X31 and received placebo or 500 ng VT per day starting at the indicated times. **(b)** Survival was monitored daily; numbers in parentheses indicate the number of mice per group. ***p < 0.001 vs. flu-alone mice. **(c)** Arterial oxygen saturation on day 5 after infection, ***p < 0.001. **(d)** Lung edema was measured by wet-to-dry ratio in mice sacrificed 5 days after infection. **(e)** Left ventricular ejection fraction on day 5 after infection was measured by echocardiography. **(f)** C57BL/6J mice were infected with mouse-adapted influenza, strain PR8 and received placebo or 500 ng VT daily starting at 48 hours after infection, *p < 0.05.

We first infected C57BL/6 mice with a lethal dose of influenza virus (Hkx31;H3N2) followed by daily intraperitoneal injection of Vasculotide (500 ng, see schematic, Fig. 1a). While no infected mice survived longer than 8 days, treatment with Vasculotide markedly improved survival even if started as late as 72 hours post-infection (80% survival if started at 24 hours after infection; 70% survival if at 72 hours, p < 0.001); treatment starting 96 hours after infection improved survival although this did not achieve statistical significance (p = 0.07; Fig. 1b); treatment with a lower dose of Vasculotide (100 ng) did not appear to be as effective (Supplemental Figure 1).

Prior to receiving Vasculotide, mice displayed typical signs of progressive illness such as arterial hypoxemia, hypothermia, and declining body weight; these features are apparent as early as 48–72 hours after infection. Vasculotide treatment started at 72 hours post-infection is able to rescue a significant subset of infected mice (Supplemental Figures 2,3). Furthermore, virus-induced hypoxemia and lung edema were greatly ameliorated with the drug (Fig. 1c–d). Echocardiography revealed that lung edema was not due to impairment of left ventricular systolic function by the virus, consistent with an increase in vascular leak from lung injury^13^; analogously, the reduction in lung edema by Vasculotide was not from an improvement in left ventricular systolic function (Fig. 1e). Of note, the drug was similarly effective against a lethal dose of PR8 (H1N1), a second strain of influenza virus that is highly virulent in mice^17^ (Fig. 1f). Next, we infected mice with the 2009 swine-origin pandemic H1N1 influenza virus. Although the mortality rate in these mice was only 25%, all of the Vasculotide-treated mice survived (Supplemental Figure 4). Thus, Vasculotide appears to be effective against lung injury caused by multiple strains of influenza.

**Figure 2:**
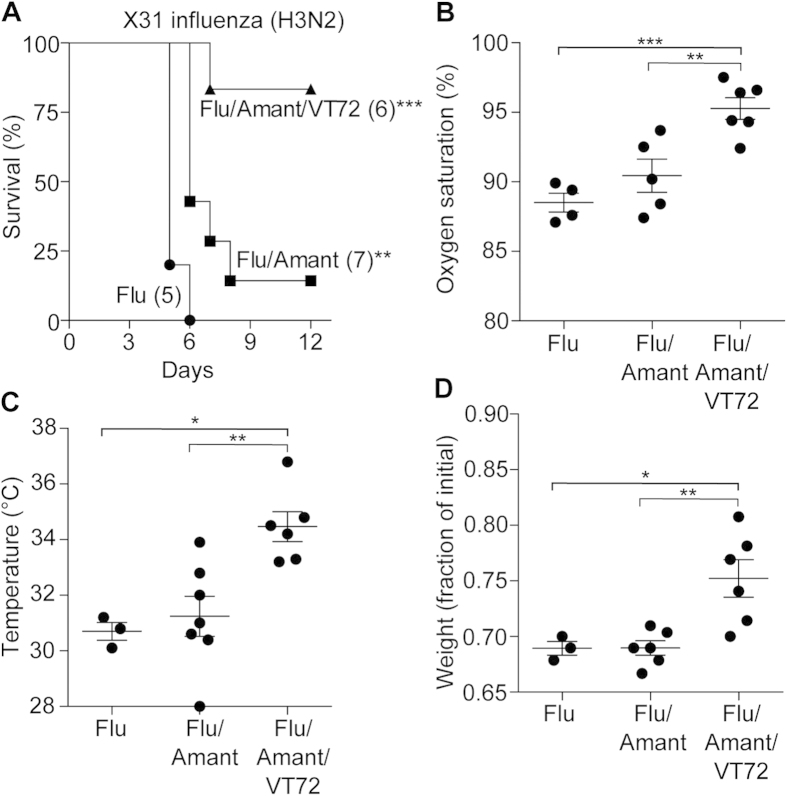
Adjuvant VT improves survival even if delayed. **(a)** C57BL/6J mice were infected with mouse-adapted influenza, strain X31 and received daily amantadine (Amant) starting at the time of infection; placebo or daily 500 ng VT was given starting 72 hours later, **p < 0.01, ***p < 0.001 vs. flu-alone mice. Numbers in parentheses indicate the number of mice per group. **(b)** Arterial oxygen saturation was measured 6 days after viral infection, **p < 0.01, ***p < 0.001 versus Flu/Amant/VT72. **(c)** Body temperature measured 5 days after infection, *p < 0.05 , **p < 0.01 vs. Flu/Amant/VT72 (**d)** Fraction of initial body weight measured 5 days after infection, *p < 0.05, **p < 0.01 vs. Flu/Amant/VT72.

In a clinical setting, an agent like Vasculotide is most likely to be administered in combination with an antiviral drug. We therefore treated influenza virus-infected mice with amantadine^18^ alone or in combination with Vasculotide. Treatment with amantadine modestly but significantly improved survival (from 0 to 14%, Fig. 2a). Adjuvant Vasculotide markedly increased survival even when started as long as 72 hours after infection (83% survival) (Fig. 2a and Supplemental Figure 5). This was in association with significant improvements in arterial hypoxemia (Fig. 2b), hypothermia and weight loss (Fig. 2c–d). These findings are especially important because mortality from influenza virus persists despite antiviral therapy and because the efficacy of such therapy declines rapidly over time^4^,^6^,^7^.

**Figure 3:**
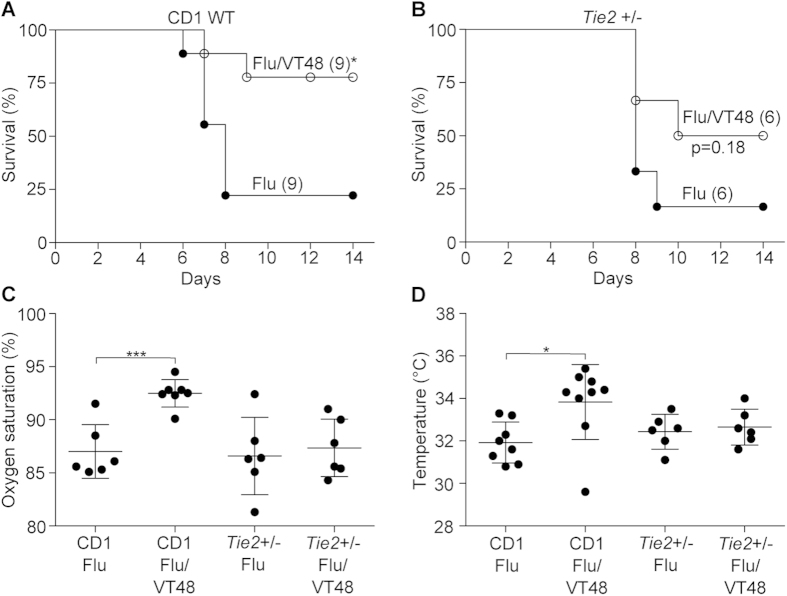
Survival benefit from VT requires Tie2. **(a)** CD1 mice were infected with influenza virus (X31) and given placebo or daily 500 ng VT starting 48 hours after infection; *p < 0.05. Numbers in parentheses indicate the number of mice per group. **(b)***Tie2*^+/−^ (bred on a CD1 background) mice were infected with influenza virus (X31) and given placebo or daily VT starting 48 hours after infection, p = 0.18. **(c)** Arterial oxygen saturation was measured 5 days after infection in CD1 and *Tie2*^+/−^ mice infected with influenza virus and administered VT, ***p < 0.001. **(d)** Body temperature measured 5 days after infection; *p < 0.05.

To prove that the benefit of Vasculotide is mediated through the Tie2 receptor, and because homozygous deletion of Tie2 is embryonically lethal^19^, we obtained haploinsufficient *Tie2*^+/−^ mice bred onto a CD1 background. These mice express significantly less Tie2 on the lung endothelium^20^. Infection of CD1 wild-type mice with influenza virus (Hkx31;H3N2) resulted in significant lethality (only 22% survival) that was greatly improved by Vasculotide (78% survival), even if started 48 hours after infection (Fig. 3a). Conversely, infection of haploinsufficient *Tie2*^+/−^ mice resulted in a similar degree of mortality (25% survival) that was not significantly changed by the drug (50% survival), although there was a trend towards an intermediate phenotype (Fig. 3b). Furthermore, in contrast to wild-type mice, Vasculotide had no effect on influenza virus-induced hypoxemia or hypothermia in the haploinsufficient mice (Fig. 3c–d). Thus, the benefit of Vasculotide is dependent on Tie2 expression.

**Figure 4:**
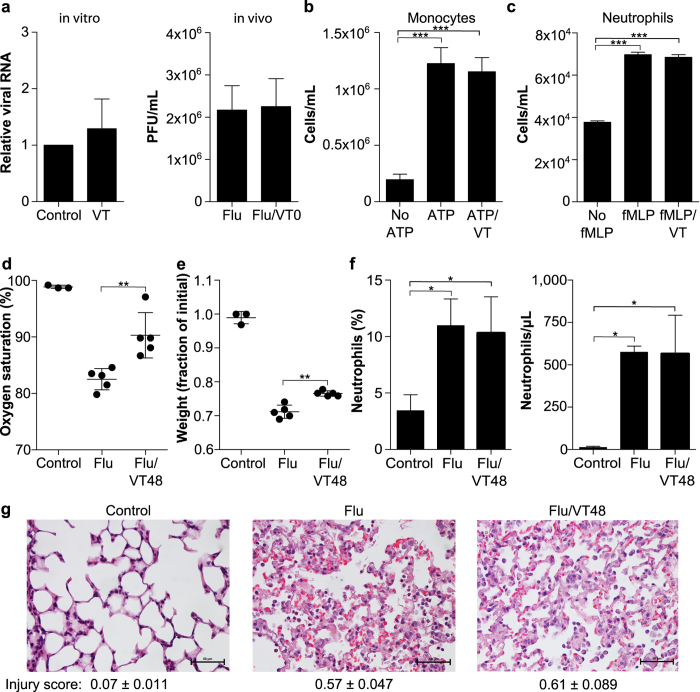
VT has no antiviral activity and does not affect innate immunity. **(a)** VT (*in vitro* 20 ng/mL, *in vivo* 500 ng) had no effect on viral replication assessed by qPCR *in vitro* or using lung homogenates for plaque-forming units (pfu) on day 5 post-infection. **(b–c)** Chemotaxis of THP1 monocyte-like cells or human neutrophils was not affected by VT, ***p < 0.001. **(d–e)** Significant effect of VT on oxygen saturation and weight loss of mice prior to histological analysis, **p < 0.01. **(f)** Influenza induces alveolar neutrophilia (day 5 post-infection) that is unaffected by VT, *p < 0.05. **(g)** Systematic scoring of acute lung injury by histology was performed in a blinded fashion. Data are mean and standard deviation from n = 5 mice in Flu and Flu/VT48 groups and n = 3 uninfected controls.

While Tie2 is widely thought to be almost exclusively expressed on endothelial cells^21^, we considered the possibility that its benefit might reflect unanticipated activity on non-endothelial tissues or on the virus itself. We first established that Vasculotide has no antiviral activity *in vitro* and *in vivo* (Fig. 4a). Second, Vasculotide did not affect chemotaxis of human and murine monocytes and neutrophils *in vitro* (Fig. 4b–c and data not shown). To our surprise, despite significantly improved arterial oxygen saturation and body weight (Fig. 4d–e), Vasculotide-treated mice exhibited no difference in the severity of lung injury determined by blinded quantification of lung histology at 5 days post-infection (Fig. 4g) (this timepoint was chosen as infected mice die soon afterwards). Concordantly, while infection induced a marked increase in alveolar neutrophil recruitment, this was unaffected by treatment with Vasculotide (Fig. 4f); alveolar and serum cytokine levels on day 5 post-infection were also unaffected (Supplemental Figure 6). Thus, the mechanism of benefit of Vasculotide appears downstream of innate immune responses and instead involves the lung endothelial barrier.

As expected, Vasculotide induced Tie2 phosphorylation and significantly attenuated thrombin-induced lung endothelial leak, as measured by electrical resistance (Supplemental Figure 7a–b). While the drug had no effect on human lung endothelial proliferation (Supplemental Figure 8), it significantly attenuated lung endothelial apoptosis *in vitro* in response to influenza virus, as assessed by cleavage of caspase-3 (Supplemental Figure 7c); we observed a similar reduction in cleaved caspase-3 in lungs from infected mice who received Vasculotide (Supplemental Figure 7d). Although the apoptotic cells in the lungs of infected mice undoubtedly reflect contributions from epithelium and leukocytes, we observed colocalization of cleaved caspase-3 with the endothelial-specific marker VE-cadherin, consistent with our *in vitro* data. Finally, while influenza infection caused loss of Tie2 from the lungs of infected mice, this was significantly attenuated by treatment with Vasculotide. Vasculotide treatment also markedly increased levels of phospho-Akt in the lung, an event that is known to be downstream of Tie2 activation^22^ and that acts as an endothelial pro-survival signal (Supplemental Figure 9).

Our findings have a number of important implications. First, these data strongly implicate failure of the lung endothelial barrier as the cause of death in murine models of severe influenza, as Vasculotide conferred a significant survival benefit against multiple strains of the virus in two strains of mice. The effect of the drug is Tie2-dependent, appears to be downstream of innate immune activation and does not require inhibition of viral replication. Second, the enhancement of lung endothelial barrier integrity did not impair leukocyte recruitment to the lung, supporting the notion that vascular leak and leukocyte transmigration may be regulated independently^23^. Accordingly, administration of Vasculotide at the time of infection (i.e. even before leukocyte infiltration could occur) was *not* harmful and still conferred great benefit (Fig. 1b). Lastly, administration of Vasculotide even 3-4 days after infection significantly improved survival, an observation with practical implications given that the moment of infection in patients is usually uncertain.

While other ligands for Tie2 have been studied as therapies for vascular leak (as reviewed in^24^), none have been reported in severe influenza virus infection and almost all required administration to be prophylactic or simultaneous with the time of infection^25^,^26^, limiting their clinical utility. The potential clinical use of recombinant angiopoietin 1, the endogenous ligand for Tie2, is limited by its tendency to bind to the extracellular matrix and to form undesirable aggregates^27^; furthermore, modified forms of the protein are reportedly difficult to synthesize and are unstable^27^. In contrast, Vasculotide was invented based on phage display experiments in which over 1 billion unique peptides were screened to identify those capable of binding with high affinity to Tie2^28^,^29^. Vasculotide bears no sequence homology to angiopoietin-1 and exhibits no activity against or binding to some 500 cellular receptors (unpublished data); its specificity for Tie2 is also strongly supported by our data using *Tie2* haploinsufficient mice. Given the existing literature on Ang1 and other putative barrier-enhancing agents^14^,^26^,^30^, the ability of delayed administration of Vasculotide to rescue mice dying of influenza (Supplemental Figure 2) is unexpected and novel. This finding, combined with its relatively low cost of production and its chemical stability, suggests that the drug might constitute a practical therapy.

Our data indicate that Vasculotide increases endothelial barrier integrity and reduces lung endothelial apoptosis, consistent with its agonism of Tie2^31^. Further work will determine whether it affects other mediators of vascular instability including angiopoietin-2^32^ and VE-PTP^33^. As its mechanism of action is independent of viral replication and instead involves modulation of the host response, it is possible that Vasculotide may not engender viral resistance, but this hypothesis will require direct testing. Since influenza virus infections are often complicated by secondary bacterial pneumonia^34^, novel agents that impair the innate immune response may not be without risk. The fact that Vasculotide does not impede neutrophil recruitment to the lung is therefore reassuring. Finally, whether the impressive benefit of Vasculotide in the therapy of the influenza virus carries implications for other infectious agents^35^ or non-infectious causes of ARDS will require further investigation.

## Methods

### Experimental design

Fourteen-week old C57BL/6J mice were purchased from Jackson Laboratories (Bar Harbor, Maine). Mice were housed in the St. Michael’s Hospital Vivarium on a standard light:dark cycle and were given free access to food and water. St. Michael’s Hospital is an accredited Biosafety Level 2 (BSL-2) facility and all mouse infection experiments were performed in a dedicated BSL-2 room in the Vivarium. Experimental procedures were conducted in accordance with the St. Michael’s Hospital Animal Care Committee guidelines for animal use and subject to an approved animal protocol (ACC297 and ACC 542, St. Michael’s Hospital).

For all experiments, mice were sedated with 5% isoflurane and infected intranasally with influenza virus (see **Virus** section, below) diluted in PBS to a final volume of 80 μL. After infection, mice were separated into weight-matched control and treatment groups; unless otherwise indicated, we used ten mice per group and mice were infected with 64 HAU of influenza virus, which caused greater than 90% mortality by day 7.

In the first experiment, we included the following groups (see Fig. 1b): mice that received the influenza virus alone (Flu) and infected mice that also received Vasculotide (500 ng in 0.1 mL PBS by intraperitoneal injection, the dose based on previous work^16^); of the latter, mice that received Vasculotide were divided *a priori* into those who received it at the time of infection (VT0) and then once daily; those that first received VT 24 hours after infection and then once daily (VT24); those that received VT starting 48 hours after infection and then once daily (VT48); those that received the drug starting 72 hours after infection and then once daily (VT72) and mice in whom the drug was started 96 hours after infection and then once daily (VT96). Control mice (Flu) received a daily intraperitoneal injection of 0.1 mL PBS. A similar experiment was performed using the PR8 subtype of influenza A and the 2009 swine-origin pandemic H1N1 influenza virus (A/Mexico/4108/2009 strain).

In other experiments to test VT as an adjuvant therapy (Fig. 2 and Supplemental Figure 5), we divided mice into the following six groups: influenza-virus infected mice (Flu); infected mice that received a dose of Amantadine by oral gavage twice daily at a concentration of 138 mg/kg/day^18^(Flu/Amant); and infected mice that received Amantadine and a daily intraperitoneal injection of VT starting 0, 24, 48 or 72 hours after infection (Flu/Amant/VT0; Flu/Amant/VT24; Flu/Amant/VT48; Flu/Amant/VT72). Control mice (Flu) received twice daily doses of sterile water by oral gavage in lieu of Amantadine.

To test the requirement for Tie2 (Fig. 3), CD-1 wildtype and *Tie2*^+/−^ mice bred onto the CD-1 background were obtained from Sunnybrook Research Institute (Toronto, Ontario); *Tie2*^+/−^ mice were backcrossed onto the CD-1 background for a minimum of 6 generations. These mice were divided into the following four groups, depicted in Fig. 3: CD-1 wildtype mice infected with influenza virus (Flu, Fig. 3a); CD-1 wildtype mice that were infected and given VT daily starting 48 hours after infection (Flu/VT48, Fig. 3a); *Tie2*^+/−^ mice infected with influenza virus (Flu, Fig. 3b); *Tie2*^+/−^ mice that were infected and given VT daily starting 48 hours after infection (Flu/VT48, Fig. 3b).

In all experiments, mice were monitored 3 times daily for the duration of the experiment (12–14 days after infection) and were scored for weight loss, hypothermia, hypoxemia, spontaneous activity (scored from 1 (moribund) to 5 (normal), as described in^36^), and other clinical features of influenza infection. Mice were euthanized if two or more of the following occurred: weight loss exceeded 30% of initial weight; temperature fell below 31 °C; the animal appeared moribund. In some experiments, mice were sacrificed on the indicated day for collection of blood and tissue samples.

### Virus

Influenza A virus HKx31 (H3N2) and A/Puerto Rico/8/34 (PR8, H1N1) were originally obtained from Dr. Tania Watts and Dr. Conrad Liles, respectively and propagated in accordance with published protocols^37^. 2009 swine-origin pandemic H1N1 influenza virus (A/Mexico/4108/2009 strain) was a generous gift from Dr. David Kelvin (University Health Network, Toronto); for this strain, mice were infected with 10^4^ EID50/mouse^38^. Viral titers from culture supernatants and murine lung homogenates were determined by performing a standard plaque-forming assay in MDCK (Madin-Darby Canine Kidney) cells or by measuring agglutination of sheep red blood cells (hemagglutinin units, HAU)^37^. *In vitro* measurement of viral replication was also measured by qPCR as previously described^39^.

### Pulse oximetry measurements

Arterial oxygen saturation was measured using the Mouse Ox Plus device and software (Starr Life Sciences, Oakmont, PA) on awake (non-anesthetized) mice using either the small or medium collar clips for C57BL/6J and CD–1 and *Tie2*^*+/−*^ mice, respectively. For C57BL/6J mice, a chemical depilatory cream was used to remove hair around the base of the neck several days before infection. For SO_2_ measurements, mice were allowed to acclimate to the collar clip for several minutes in their home cage before recording the maximal SO_2_ measurement.

### Detection of neutrophils in bronchoalveolar lavage fluid (BALF)

HKx31-infected C57BL/6J mice treated with or without VT starting 48 hours after infection were sedated and sacrificed 5 days after infection by cardiac puncture. This timepoint was chosen as infected mice die soon afterwards. After sacrifice, the trachea was exposed and the lungs were inflated with 0.8 mL cold PBS with 0.6 mM EDTA. After 30 s, 0.7 mL BALF was recovered and centrifuged at 300x g to pellet the cells. Supernatant was collected and immediately frozen for analysis of BALF cytokines. The BALF pellet was resuspended in 300 μL PBS. Neutrophils were stained with 0.1 μL of PerCP/Cy5.5 anti-mouse CD14 (BioLegend, San Diego, CA) for 20 minutes prior to sorting and counting on the Miltenyi MACSQuant (Meltenyi Biotec, San Diego, CA) analyzer.

### Analysis of serum and BALF cytokines

Plasma was isolated from whole blood 5 days after HKx31 infection in C57BL/6J mice treated with or without VT starting 48 hours after infection. Plasma and BALF samples were run on a custom ProcartaPlex™ (Procarta, San Diego, CA) cytokine array featuring the following analytes: IL-1 β, IL-6, IL-10, IFN- ϒ, TNF- α, MCP-1, RANTES, VEGF, MIP-1 α, IL-15. Samples were prepared based on manufacturer’s instructions and run on a BioPlex**®** Multiplex system (Bio-Rad, Mississauga, ON).

### Measurement of edema

C57BL/6J mice were infected with HKx31and VT (500 ng) or PBS was injected by IP injection once daily for 5 days. On day 5, mice were sacrificed by cardiac puncture of the right ventricle. The circulatory system was flushed with 10 mL cold PBS by instillation through the left ventricle. The entire right lung was removed, patted dry, and then weighed for determination of wet weight. Lung tissues were dried for 24 hours and then weighed for the dry weight. Degree of lung edema is expressed as the wet-to-dry ratio of lung weights.

### Echocardiography

C57BL/6J mice were infected with HKx31 influenza and VT (500 ng daily) or PBS treatment was started 48 hours after infection. On day 4, mice were lightly sedated and a depilatory cream was used to remove hair from the ventral surface of the thorax and abdomen. On day 5, mice were lightly sedated and left ventricular function was measured by echocardiography as previously reported^40^.

### Histology and immunohistochemistry

HKx31-infected C57BL/6J mice (n = 5 per group and 3 uninfected controls) treated with or without VT starting 48 hours after infection (Flu/VT48) were sedated and sacrificed by cardiac puncture 5 days after infection. The lungs were inflated with and then submerged in 10% buffered formalin for 3 days before tissue processing. Lung tissues were embedded in paraffin, sectioned in serial 4 μm sections, and mounted on slides. Slides were stained with hematoxylin and eosin; a lung pathologist (D.H.) blinded to the experimental design scored acute lung injury based on consensus criteria, yielding a composite score between 0 (no injury) to 1 (highest degree of lung injury)^41^. Degree of lung injury was determined by scoring 20 randomly selected high power fields (40x magnification) per mouse. For detection of cleaved caspase-3, immunofluorescence microscopy was performed on formalin-fixed paraffin embedded lung tissue as previously described^42^. Sections were dual-stained with a goat polyclonal antibody directed against VE-cadherin (Santa Cruz Biotechnology) and a rabbit polyclonal antibody directed against cleaved caspase-3 (Cell Signaling Technology). Slides were washed and subsequently incubated with Alexa Fluor 555 donkey anti-goat and Alexa Fluor 488 donkey anti-rabbit secondary antibodies, both from Life Technologies Inc. (Burlington, ON, Canada). Blinded quantification of cleaved caspase-3 was performed on 10 randomly selected lung fields per mouse acquired by spinning disc confocal microscopy (Zeiss Axiovert 200 M microscope, settings kept constant between conditions) and analyzed using ImageJ (NIH).

### Cell culture and reagents

Primary human lung microvascular endothelial cells (HPMECs) obtained from PromoCell (Heidelberg, Germany) were cultured in EBM-2 media (Lonza, Basel, Switzerland) with the recommended supplements and used in passages 6–7. The THP-1 human monocytic cell line was a gift from Amira Klip (Toronto). Human neutrophils were obtained from healthy volunteers and were isolated from whole blood by density gradient separation^43^. Vasculotide, a PEGylated tetrameric peptide agonist of the Tie2 receptor, was synthesized as previously described^16^ and prepared to a concentration of 50 ng/μL in sterile phosphate-buffered saline (PBS) without calcium and magnesium. A working solution of VT was prepared by diluting the stock ten-fold in PBS.

### Immunoblot

HPMEC-L were grown to 90% confluency on 6-well plates and treated with X31 influenza or X31 influenza plus 20 ng/nL VT for 24 hours. Lysates were prepared with lysis buffer (62.5 mM Tris-HCl pH 6.8, 2% SDS, 10% glycerol, 10 mM DTT). SDS page was performed on a 12% polyacrylamide gel. Immunoblotting was performed as previously described^44^ with the exception of the blocking step, which used 5% bovine serum albumin in TBS instead of 5% milk. In some experiments, lung homogenates were generated from infected mice as follows: after euthanasia, the circulation was flushed with 10 mL ice-cold PBS. The lungs were homogenized in buffer containing 137 mM NaCL, 2 mM EDTA, 10% glycerol, 1% Triton X-100, 20 mM Tris-HCL, 5 mM Sodium orthovanadate, and complete protease inhibitor cocktail tablets and separated using 8% polyacrylamide gels. Proteins were transferred to nitrocellulose membranes, blocked for 1 hour in 5% milk in TBS, and probed overnight with primary antibody at 4 °C. After washing, blots were incubated with HRP-conjugated secondary antibodies for 1 hour, washed, and then visualized by chemiluminescence (ThermoScientific). Band intensity was quantified using ImageJ (NIH) and normalized to the loading control after background correction. The anti-phospho-Akt (Ser473) antibody was from Cell Signaling; the anti-Tie2 (C20) antibody was from Santa Cruz; all other antibodies were from Santa Cruz Biotechnology.

### Chemotaxis assay

2 × 10^5^ neutrophils (isolated from healthy human volunteers) in 100 μL of DMEM with low-glucose or 2 × 10^5^ THP-1 cells in 100 μL of RPMI 1640 with 10% FBS and 1% penicillin/streptomycin containing 20 ng/ml or 100 ng/ml VT or PBS were seeded into the upper part of a Transwell chamber (8-μm pore size, 6.5 mm in diameter; Corning Life Sciences). The lower chamber of the Transwells was filled with 300 μL of DMEM containing vehicle (DMSO) or 1 μM fMLP for neutrophil chemotaxis or with 300 μL of RPMI 1640, 10% FBS, 1% penicillin/streptomycin containing 10 μM ATP for THP-1 chemotaxis. After 4 h of incubation at 37 °C, the migrated neutrophils or THP-1 cells were recovered from the lower chamber and counted using a hemocytometer.

### Electric cell substrate impedance sensing (ECIS)

An ECIS Ztheta system (Applied Biophysics, Troy, NY) was used to follow barrier function. HPMECs were seeded on Applied Biophysics ECIS culture arrays pretreated with 10 mM cysteine at 4.0 × 10^4^/well and grown for 48 h to allow the establishment of a confluent layer. Next, the arrays were placed into the ECIS system and impedance and capacitance (C) data were collected (and R values calculated by the software) continuously using the frequency scan mode. After obtaining a baseline, the measurement was paused and thrombin was added to the arrays in 20 μl medium to induce endothelial permeability, at a final concentration of 1 unit/mL. Some wells received VT alone or in combination with thrombin at a concentration of 20 ng/mL. Controls received 20 μl medium without thrombin. For each condition measurements were performed in duplicate. Curves were normalized to the resistance level prior to thrombin administration using the ECIS software. The ECIS trace represents the data collected at 500 Hz and is representative of 5 independent experiments.

### MTT and scratch assay

HPMECs were seeded on 96 well tissue culture plates at a density of 3.0 × 10^4^ cells/mL (8 wells per condition). Cells were incubated for four hours to allow cells to attach. After 4 hours (time 0), the media was changed to EGM-2 (control) or EGM-2 with 20 ng/mL VT. For baseline and endpoint measurements, MTT (Sigma) was added to the cells at time 0 and 24 hours after treatment followed by a 2-hour incubation. MTT and media were aspirated and the cells were solubilized in 100 uL DMSO and shaken for 5 minutes. Absorbance was measured at 538 nm.

For the scratch assay, HPMECs were grown to confluency on 12-well tissue culture plates marked on the bottom with an open square to define the scratch area. A single scratch was made through the scratch area using a sterile p1000 pipette tip. The initial scratch area was imaged by phase-contrast microscopy. Cells were washed once with media and then treated with EGM-2 or EGM-2 with 20 ng/mL VT for 24 hours. After 24 hours the cells were imaged again to assess the degree of wound healing. Phase-contrast images were exported to ImageJ for quantification of baseline and endpoint wound areas. Wound healing is expressed as a fraction of the endpoint area occupied by cells over the baseline scratch area. Quantification of wound healing was performed in a blinded fashion.

### Statistics

All statistics were performed using GraphPad Prism (La Jolla, CA) software. Comparisons between two groups were made using unpaired, two-tailed Student’s t-tests with statistical significance set at P < 0.05. Comparisons of more than 2 groups were made using one-way ANOVA with post-hoc analyses applying Bonferroni’s adjustment for comparisons between selected groups. For comparing Kaplan-Meier survival curves, the Log-rank test was used with P < 0.05. Statistics for ECIS experiments were conducted with Repeated Measures ANOVA and Sidak’s test for multiple comparisons.

## Additional Information

**How to cite this article**: Sugiyama, M. G. *et al*. The Tie2-agonist Vasculotide rescues mice from influenza virus infection. *Sci. Rep*. **5**, 11030; doi: 10.1038/srep11030 (2015).

## Supplementary Material

Supplementary Information
