# Supplementary material for: The Tie2-agonist Vasculotide rescues mice from influenza virus infection
Source: Sci Rep. 2015 Jun 5;5:11030. doi: 10.1038/srep11030 (PMC4457136; doi:10.1038/srep11030)
Supplement: Supplementary Information [file srep11030-s1.pdf]

The Tie2-agonist Vasculotide rescues mice from influenza virus infection

Mr. Michael Sugiyama , Dr. Susan Armstrong , Ms. Changsen Wang , Dr. David Hwang , Dr. Howard Leong-Poi , Dr. Andrew Advani , Ms. Suzanne Advani , Dr. Haibo Zhang , Dr. Katalin Szaszi , Mr. Arata Tabuchi , Dr. Wolfgang Kuebler , Dr. Paul Van Slyke , Dr. Dan Dumont, Dr. Warren L. Lee

Legends to Supplementary Figures

Supplemental Figure 1. Effect of a lower dose of VT on survival. Mice were infected with influenza A and received a lower dose of Vasculotide (VT; 100 ng by IP injection) or control starting 48 hours after infection and then once daily. The numbers in parentheses indicate the numbers of mice in each group. P=0.19 for the comparison between the two groups.

Supplemental Figure 2. VT administered 3 days after infection rescues mice from influenza. Clinical parameters of infected mice (n=5 per group) prior to and after Vasculotide (VT72; started 72 hours post-infection, arrow). Data are mean and standard error. For clarity, mice receiving VT on a different schedule are shown in the next figure. **(a)** Oxygen saturation, **(b)** Temperature **(c)** Activity score, **(d)** Body weight.

Supplemental Figure 3. VT protects and rescues mice from influenza infection. Clinical parameters of infected mice (n=10 per group) prior to and after Vasculotide was started. VT0: started at the time of infection; VT24: started 24 hours after infection; VT48: started 48 hours after infection. Data are mean and standard error. **(a)** Body weight **(b)** temperature, **(c)** Activity score.

Supplemental Figure 4. Effect of VT against swine-origin pandemic H1N1 influenza. Mice were infected with swine-origin pandemic H1N1 influenza and received Vasculotide (500 ng) or control starting 48 hours after infection (arrow) and then once daily; n=8 mice per group; **(a)** Survival curve, p=0.14 for comparison of the two groups. **(b)** Body temperature **(c)** Weight **(c)** Activity.

Supplemental Figure 5. Adjuvant VT improves survival. Adjuvant VT (compared to Amantadine alone, Amant) improves survival even if started 24 or 48 hours after infection. The number in parentheses indicates the number of mice in each group. **p<0.01, ***p<0.001 versus influenza-only infected mice

Supplemental Figure 6. VT does not affect cytokine levels. Serum **(a)** and alveolar lavage **(b)** cytokine levels on day 5 post-infection are largely unaffected by VT (500 ng) started 48 hours after infection, *p<0.05.

Supplemental Figure 7. VT increases endothelial electrical resistance and reduces apoptosis. **(a)** Tie2 phosphorylation in human lung microvascular endothelium increases at 15-30 minutes after stimulation with VT 20 ng/mL; *p<0.05. **(b)** Transendothelial electrical resistance drops after stimulation with thrombin but is increased by VT 20 ng/mL as measured by electrical cell substrate impedance sensing (ECIS); ****p<0.0001 for control vs. thrombin,

Mr. Michael Sugiyama , Dr. Susan Armstrong , Ms. Changsen Wang , Dr. David Hwang , Dr. Howard Leong-Poi , Dr. Andrew Advani , Ms. Suzanne Advani , Dr. Haibo Zhang , Dr. Katalin Szaszi , Mr. Arata Tabuchi , Dr. Wolfgang Kuebler , Dr. Paul Van Slyke , Dr. Dan Dumont, Dr. Warren L. Lee

control vs. VT, VT vs. thrombin+VT. Tracing is representative of 5 experiments. **(c)** Primary human lung microvascular endothelial cells were exposed to influenza (X31, multiplicity of infection 1) for 24 hours, with or without 20 ng/mL VT, and lysates were probed for cleaved caspase-3. Quantification at right-hand panel; *$p<0.05$. **(d)** Lung sections from mice on day 5 post-infection (some of which received VT starting 48 hours post-infection) were probed for cleaved caspase-3 (green), VE-cadherin (red) and nuclei which were stained with DAPI (blue). Arrows indicate apoptotic cells; colocalization with VE-cadherin projects as yellow. Blinded quantification is shown in the right-hand panel; *$p<0.05$. n=5 for Flu and Flu/VT48 groups, n=3 uninfected controls.

Supplemental Figure 8. VT does not affect lung endothelial proliferation. Proliferation of primary human lung microvascular endothelium alone or in response to VT 20 ng/mL was assessed by MTT assay (left) or scratch assay (right), both after 24 hours.

Supplemental Figure 9. VT prevents influenza virus-induced loss of Tie2 and induces Akt phosphorylation in the lung. Mice were infected with influenza A and received VT (500 ng) or control starting at 48 hours after infection and then once daily. Four days after infection, mice were euthanized and lungs were harvested 30 minutes (one mouse) or 60 minutes (3 mice) after VT injection. Lung homogenates were probed as indicated. Bottom histogram shows expression level of Tie2 in the lung; *$p=0.04$ for the comparison between VT-treated and control-treated influenza-infected mice.

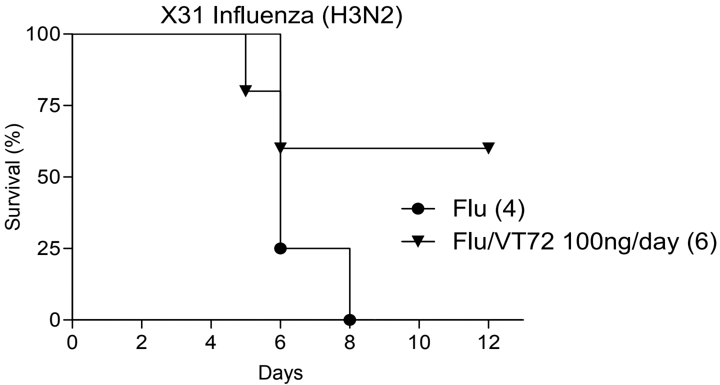

Figure S1. Effect of a lower dose of VT on survival.

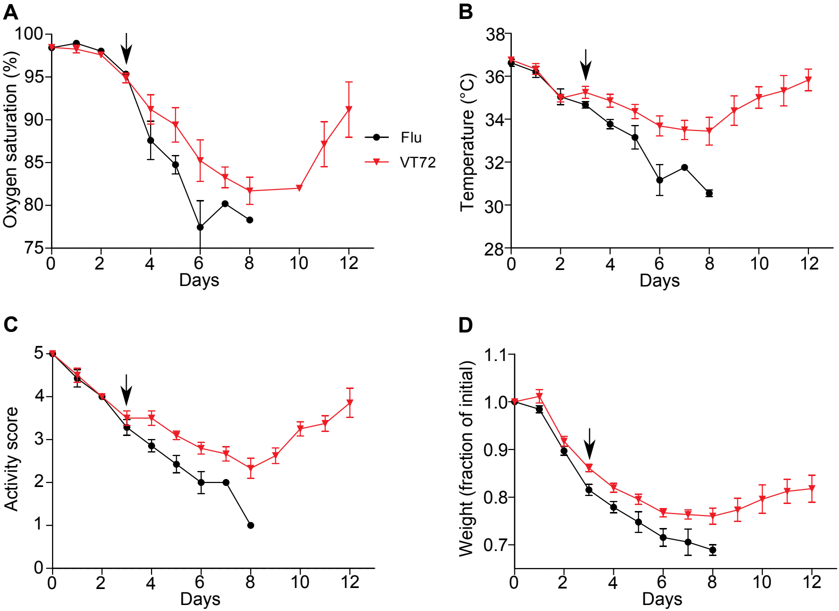

Fig. S2. VT administered 3 days after infection rescues mice from influenza.

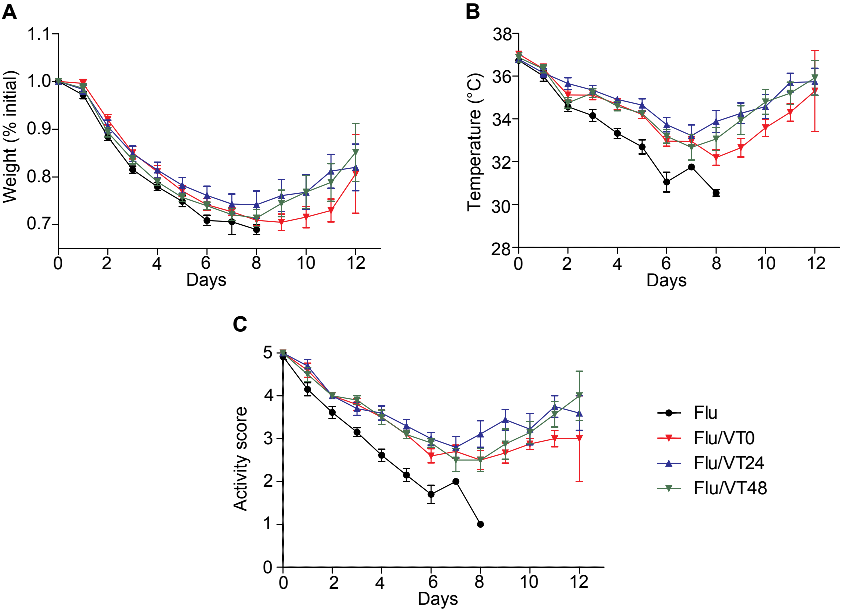

Fig. S3. VT protects and rescues mice from influenza infection.

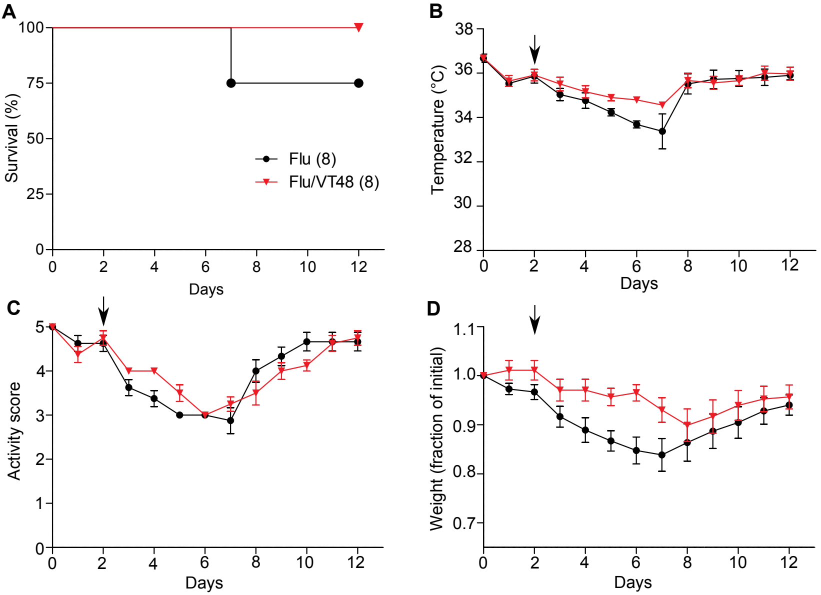

Fig. S4. Effect of VT against swine-origin pandemic H1N1 influenza.

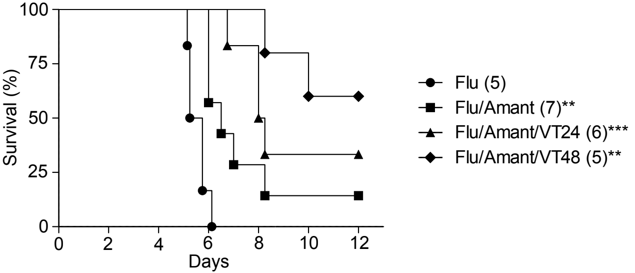

Fig. S5. Adjuvant VT improves survival.

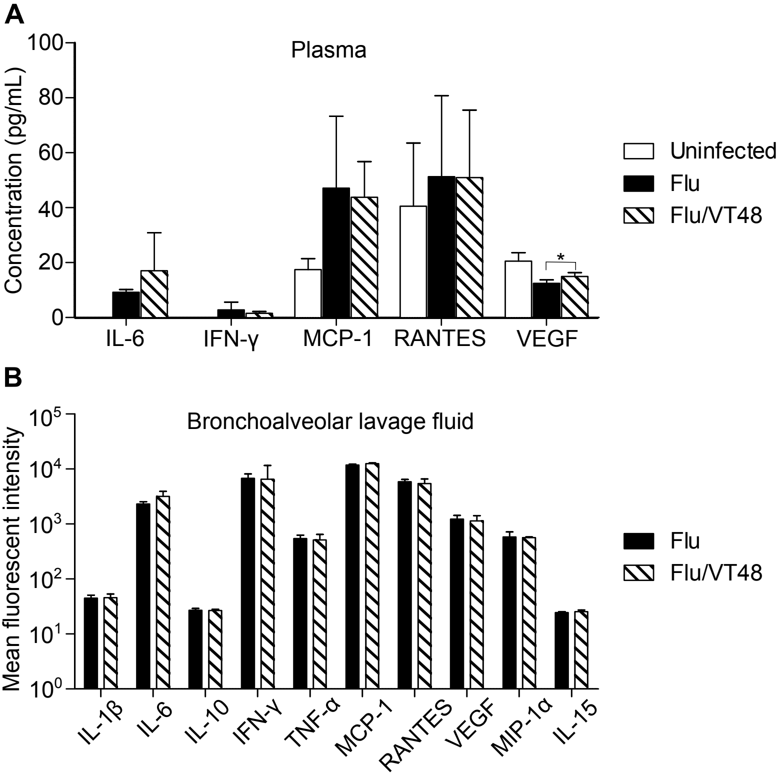

Fig. S6. VT does not affect cytokine levels.

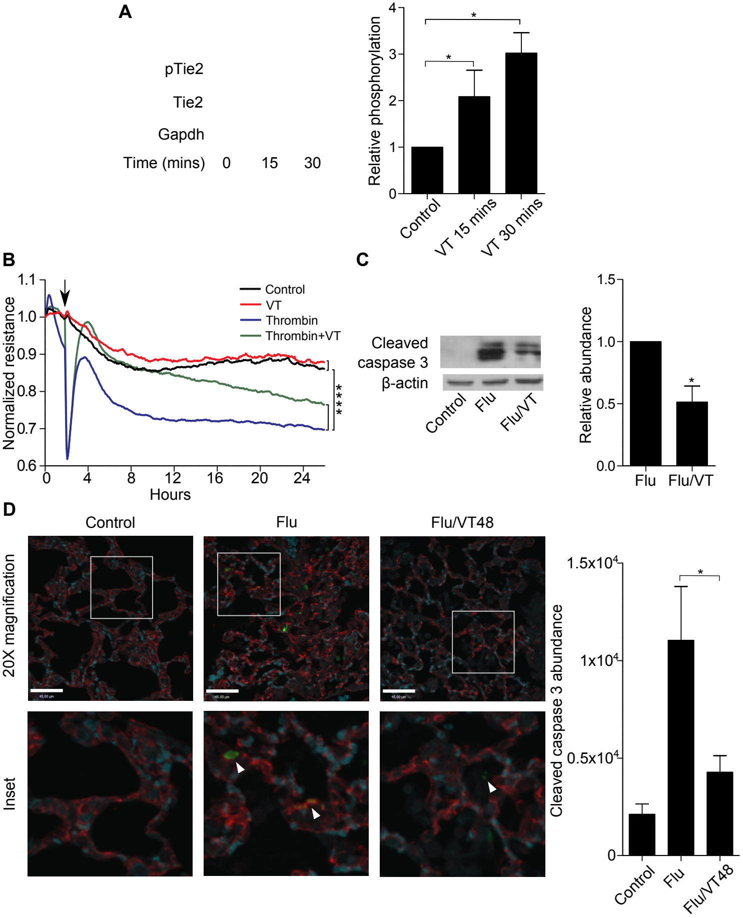

Fig. S7. VT increases endothelial electrical resistance and reduces apoptosis.

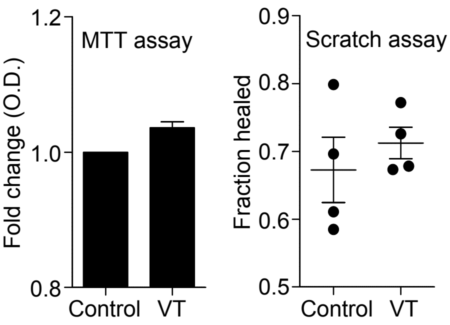

Fig. S8. VT does not affect endothelial cell proliferation

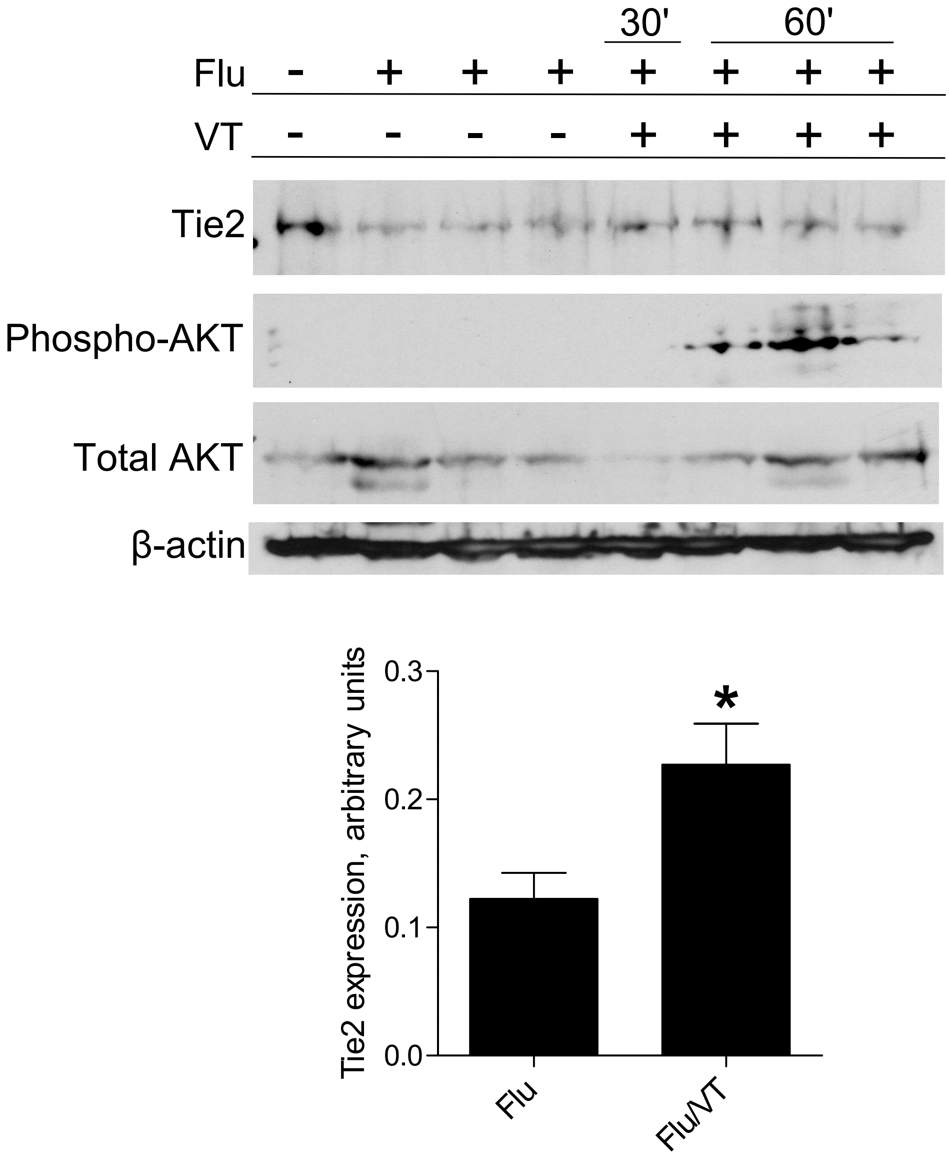

Fig. S9. VT prevents influenza virus-induced loss of Tie2 and induces AKT phosphorylation in the lung.
